# The effect of exercise on sleep habits of children with type 1 diabetic: a randomized clinical trial

**DOI:** 10.1186/s12887-024-04760-9

**Published:** 2024-04-27

**Authors:** Nastaran Amiri, Kimia Karami, Fatemeh Valizadeh, Yaser Mokhayeri

**Affiliations:** 1 Department of Pediatrics Nursing, School of Nursing and Midwifery, Lorestan University of Medical Sciences, Khorramabad, Iran; 2 Social Determinants of Health Research Center, School of Nursing and Midwifery, Lorestan University of Medical Sciences, Khorramabad, Iran; 3 Razi Herbal Medicines Research Center, Department of Pediatrics Nursing, School of Nursing and Midwifery, Lorestan University of Medical Sciences, Khorramabad, Iran; 4 Cardiovascular Research Center, Shahid Rahimi Hospital, Lorestan University of Medical Sciences, Khorramabad, Iran

**Keywords:** Exercise, Sleep habits, Children, Type 1 diabetes

## Abstract

**Background:**

Adequate sleep and exercise are important components of the human lifestyle. Paying attention to these two factors is very important to improve the condition of children with type 1 diabetes. Therefore, this study aimed to investigate the effect of exercise on sleep habits in children with type 1 diabetes.

**Material & methods:**

62 children with type 1 diabetes participated in this clinical trial. They will be divided into the intervention group (31) and the control group (31). Sleep habits were measured using the Children’s Sleep Habits Questionnaire (CSHQ). All children’s parents completed the CSHQ. The intervention for the experimental group consisted of 8 weeks of regular exercise program. The exercise program was prepared as an educational video and provided to parents. Paired sample t-test and ANCOVA test were used with SPSS 23.

**Results:**

62 children with an average age of 9.32 ± 2.02 were studied. Fifty-four and eight% of the children were girls and the rest were boys. The analysis of the variance test showed a significant difference (F = 144.72, *P* ≤ 0.01) between the average score of the sleep habits of the control group (62.45 ± 5.12) and the experimental group (47.06 ± 4.39).

**Conclusion:**

Sleep habits in the experimental group improved after 8 weeks of exercise training using educational videos. Exercise as a non-pharmacological treatment is an effective way to manage diabetes and improve sleep quality in diabetic children.

## Background

Type 1 diabetes (T1D) is one of the most common endocrine diseases in children, and its incidence has been increasing in the last two decades [1]. The average annual incidence of T1D in Iran is estimated to be 3.7 cases per one hundred thousand people. In 2021, the global number of people with diabetes is reported to be 8.4 million, and it is predicted to reach 13.5 to 17.4 million by 2040 [2–4]. One of the problems of diabetic patients is sleep disorders. Sleep is based on physiological and mental processes and is important in promoting health [5]. According to studies, sleep disorder is one of the complications of diabetes and can lead to an increase in blood sugar [6, 7]. To improve sleeping habits in diabetic patients, some experimental evidence has shown that non-pharmacological treatments are the best way to improve sleeping habits due to balance and improvement of energy in the body, as well as promoting health and relaxation in the person [8]. Exercise is one of the non-pharmacological interventions. In this regard, the results of a systematic review show that exercise can improve sleep habits without having negative effects [9]. Exercise can also have a positive effect on the quantity of sleep. People who exercise regularly sleep longer than those who do not exercise [10].

Exercise and regular physical activities are vital components in changing the lifestyle, prevention, and management of diabetes and they play a very important role in controlling blood sugar, reducing cardiovascular complications, weight control, and improving the mental and physical health of children [11, 12]. The findings of some studies indicate that exercise can benefit diabetic patients by reducing inflammation and improving antioxidant defense [13], blood glucose regulation, and lipid profile [14].

As mentioned, one of the complications of T1D is a sleep disorder. Therefore, exercise therapy and other treatments can be used to prevent and treat many complications of this T1D [15]. Exercising children is not only a hobby for them but is also studied as a tool to improve their health [16]. The spread of infectious diseases such as Covid-19 has limited the presence of children in sports clubs and crowded places such as parks. Using educational videos of sports exercises should be an effective solution to overcome this challenge. In many studies on adults, exercise has been confirmed as an effective way to improve sleep habits. However, the studies conducted on the sleep habits of children with type 1 diabetes and the role of physical activities in improving the quality of sleep of these children are relatively limited. Therefore, the present study aims to investigate the effect of exercises using educational videos on the sleeping habits of children with T1D.

## Materials and methods

This study was conducted on children with T1D referred to the clinics of Mohammad Kermanshahi and Taleghani Kermanshah teaching hospitals in western Iran from December 2021 to January 2023. This study was registered in Iran’s clinical trial site with the number IRCT20221112056480N1, (29-10-2021).

The sample size was calculated based on the statistical indicators reported in Wan Yunus’s study [17] and using G*power sample size software. Considering an effect size of 0.87, an alpha coefficient of 0.05, and a test power of 90%, the required sample size of 31 children with T1D in each group and a total of 62 eligible samples were randomly selected in 2 equal groups (31 in each group). The samples were assigned to two groups using the permuted block randomization method. The randomization unit was children and the block size was 4. This study followed the CONSORT 2010 guidelines for reporting randomized controlled trials.

The entry criteria were: children ranging in age from 6 to 12 years, having a history of diabetes for one year or more, the residence of the child’s parents in Kermanshah city, having the literacy of the child’s parents, having a smartphone, not having mental and skeletal-muscular disease, not participating in another diabetes-related educational program. Exclusion criteria included: non-activity in a professional sport, non-completion of the questionnaire, children who have a disease other than diabetes, children who take drugs that affect blood sugar, and children who have autoimmune diseases that affect diabetes.

The data collection tool included a demographic profile questionnaire and a children’s sleep habits questionnaire (CSHQ). This questionnaire has 33 items and 8 subscales including bedtime resistance, sleep onset delay, sleep duration, sleep anxiety, night wakings, parasomnias, sleep-disordered breathing, and daytime sleepiness. The scoring range of the questions is from 1 (rarely) to 3 (always) and the total marks are from 33 to 99. High scores indicate the unfavorable state of sleeping habits in children. The CSHQ has good reliability with high internal consistency (0.56 until 0.93) as well as test-retest reliability (0.62 until 0.79). The present study evaluated the internal correlation using Cronbach’s alpha coefficient of 0.77.

### Data gathering

Lorestan University of Medical Sciences Ethics Committee granted ethical approval for the study (IR.LUMS.REC.1401.174, 2022-10-19) and the Declaration of Helsinki maintained the study protocol. After obtaining permission, a training session was held for the mothers of children in the intervention group to explain the objectives of the study. According to the type of membership of the mothers in one of the virtual social networks (including Telegram, WhatsApp, or the internal social network with the name Shad), the exercise training video was sent to the mothers. In addition to sending the video, the exercise program was also sent to the mothers. Mothers were suggested to accompany their children while watching the video. The exercise program was designed with the opinion of a physical education specialist. In the design of exercises, criteria including intensity of exercises, enjoyment, and safety were considered [18, 19].

The recorded video was edited using TechSmith Camtasia Studio software and its sounds and extra parts were removed. The prepared video was sent to mothers using virtual social networks. In terms of time, the sports training protocol consisted of 8 weeks. There were 3 training sessions per week and each session consisted of 17 to 40 min. The type of exercise included slow walking and fast walking. Each training session was performed in three time periods including warm-up, fast walking, and cool-down (Table 1).

To effectively perform sports exercises by children, recommendations were given to mothers: (1) If they see signs of weakness and lethargy in their children, they should stop exercising and prepare them to do exercises after recovery. (2) It was emphasized that the children’s routine meals and snacks should remain unchanged. (3) They were advised to provide their children with food containing appropriate carbohydrates and calories if they noticed symptoms of hypoglycemia during exercise. (4) It was emphasized that the exercise program should be done at a specific time, for example, in the evening. (5) Mothers were asked to measure their children’s blood sugar using a Glucose meter (glucometer) before exercise and after exercise. (6) The mothers were requested to keep a record of the insulin regimen program during the intervention and share the results with the researcher.

**Table 1:** Exercise activity program for eight weeks

Week	Warm-up time	Fast walking time	Cool-down time	total time
First week	Slow walking (5 min)	Fast walking (7 min)	Slow walking (5 min)	17 min
Second week	Slow walking (5 min)	Fast walking (10 min)	Slow walking (5 min)	20 min
Third week	Slow walking (5 min)	Fast walking (14 min)	Slow walking (5 min)	24 min
Fourth week	Slow walking (5 min)	Fast walking (17 min)	Slow walking (5 min)	27 min
Fifth week	Slow walking (5 min)	Fast walking (20 min)	Slow walking (5 min)	30 min
Sixth week	Slow walking (5 min)	Fast walking (22 min)	Slow walking (5 min)	32 min
Seventh week	Slow walking (5 min)	Fast walking (26 min)	Slow walking (5 min)	36 min
Eighth week	Slow walking (5 min)	Fast walking (30 min)	Slow walking (5 min)	40 min

### Statistical analysis

Data was analyzed using a statistical package for the social sciences (SPSS version 23). The normality of the data was evaluated using the K-S test. The result of the test showed that the data are normally distributed. Independent t-test and chi-square test were used to check the demographic data. Paired t-test was used to compare the average score of each group in the pre-test and the post-test. Analysis of variance test was used to check the difference between the average of the control and intervention groups. Alpha was set at 0.05.

## Results

The results showed all individual characteristics were homogeneous in both control and experimental groups (Table 2). 68% of the children were managed with one basal insulin injection and one mealtime insulin injection. The rest of the participants (32%) used 2 basal insulin injections and 1 mealtime insulin injection for diabetes control. Table 3 shows the results of statistical analysis related to sleep habits and its subscales in both control and experimental groups in the pre-test and post-intervention stages. The results of the paired t-test showed that there is no statistically significant difference between the average score of sleep habits and its subscales in the pre-test and post-test stages in the control group (*P* > 0.05). In the pre-test stage, no statistically significant difference was found between the score of sleep habits of the control group and the experimental group (*P* > 0.05). A statistically significant difference was found between the average scores of the children of the experimental group in the pre-test and post-test stages (*P* < 0.05) (Table 3).

**Table 2:** Comparison of socio-demographic characteristics of the studied subjects in both groups

Variable		Group (Mean ± SD)		*P*-value
		Control	Experiment	
Age (Child)		9.35 ± 1.79	9.30 ± 2.24	0.92
Hight (Child)		140/61 ± 16.28	138.66 ± 17.36	0.25
Weight (Child)		34.51 ± 12.18	34.45 ± 17.20	0.98
Father’s age		46.96 ± 5.66	42.03 ± 5.85	0.96
Mother’s age		36.90 ± 5.44	37.22 ± 5.37	0.81
Variable		Group (N, Percentage)		*P*-value
		Control	Experiment	
Sex	Boy	16 (51.00%)	12 (39.00%)	0.307
	Girl	15 (49.00%)	19 (61.00%)	
Age range	6–8	10 (32.25%)	13 (42.00%)	0.372
	9–11	16 (51.61%)	8 (26.00%)	
	12	5 (16.12%)	10 (32.00%)	
Education of father	Elementary	1 (3.22%)	0	0.308
	Secondary	2 (6.45%)	8 (25.80%)	
	Diploma	10 (32.25%)	9 (29.03%)	
	Bachelor	15 (48.38%)	12 (38.70%)	
	Master	2 (6.45%)	2 (6.45%)	
	Ph.D.	1 (3.22%)	0	
Education of mother	Elementary	4 (12.90%)	4 (12.90%)	0.140
	Secondary	0	6 (19.35%)	
	Diploma	9 (29.03%)	8 (25.80%)	
	Bachelor	17 (54.83%)	12 (38.70%)	
	Master	1 (3.22%)	1 (3.22%)	

**Table 3:** Results of paired t-test and analysis of covariance (covariance) analysis of sleep habits and its sub-scales in both control and experimental groups

Variable		Pre-test	Post-test	Paired Sample T-Test		ANCOVA	
				t	*P*-value	F	*P*-value
Bedtime resistance	Control	12.83 ± 2.84	11.77 ± 1.89	1.83	0.07	40.81	0.01
	Experiment	11.64 ± 2.56	8.51 ± 1.98	5.14	0.01		
Sleep onset delay	Control	1.70 ± 0.68	1.51 ± 0.81	-0.96	0.235	11.41	0.01
	Experiment	1.77 ± 0.80	1.32 ± 0.70	2.13	0.04		
Sleep duration	Control	4.56 ± 1.24	4.87 ± 1.26	-0.99	0.627	3.31	0.01
	Experiment	4.70 ± 1.37	3.80 ± 1.16	2.69	0.01		
Sleep anxiety	Control	8.22 ± 2.14	7.74 ± 2.33	0.86	0.39	24.36	0.01
	Experiment	8.32 ± 1.72	5.41 ± 1.25	7.35	0.01		
Night wakings	Control	5.22 ± 1.05	4.83 ± 1.34	1.26	0.209	5.45	0.01
	Experiment	5.58 ± 1.52	4.06 ± 1.15	3.85	0.01		
Parasomnias	Control	12.00 ± 1.86	11.90 ± 2.76	0.16	0.063	29.32	0.01
	Experiment	13.77 ± 1.96	8.70 ± 1.44	11.92	0.01		
Sleep-disordered breathing	Control	4.80 ± 1.35	4.38 ± 1.38	1.20	0.232	1.07	0.30
	Experiment	5.74 ± 1.48	4.06 ± 1.06	5.09	0.01		
Daytime sleepiness	Control	15.29 ± 2.97	15.03 ± 2.77	0.53	0.59	35.51	0.01
	Experiment	15.83 ± 2.85	11.16 ± 1.80	7.86	0.01		
Sleep habits	Control	64.41 ± 7.98	63.38 ± 5.12	1.15	0.253	144.72	0.01
	Experiment	73.29 ± 6.70	47.06 ± 4.39	17.96	0.01		

There is a statistically significant difference between the average score of sleeping habits in the experimental and control groups (F = 144.72, *P* < 0.01). Among the sleep habits subscales, there was no significant difference between the mean of the two groups only in the breathing disorder subscale (F = 1.07, *P* = 0.30) (Table 3). A significant difference was found (*P* ≤ 0.05) between the mean blood sugar of children in the intervention group before (155.38 ± 40.43) and after exercise (124.03 ± 32.25).

## Discussion

In this study, designed exercises showed a positive and significant effect on improving sleeping habits. This result is in line with some other research results. According to a study, yoga and aerobics can enhance sleep quality for individuals with diabetes [20]. Another study showed that high-intensity interval training improves the sleep quality of individuals with type 1 diabetes [21]. The study by Sharma et al. demonstrated the significant impact of Pilates-based mat exercises on sleep quality and overall quality of life for diabetic patients [22].

Exercise and physical activity can improve sleep habits in diabetic patients by increasing the non-rapid eye movement sleep phase, decreasing the rapid eye movement phase, and reducing the sleep latency period [23, 24]. Evidence indicates that individuals with type 1 diabetes experience reduced sleep duration, which results in peripheral insulin resistance [25]. Therefore, selecting the appropriate time, intensity, and type of exercise training can enhance the sleep quality of individuals with diabetes [24].

Sleep resistance is one of the subscales of sleep habits. The findings of this study showed that the average score of this subscale in the children of the experimental group after the intervention was significantly lower than the children of the control group. This finding indicates the effect of exercise training on improving sleep resistance. Evidence from other studies shows that exercise can be a useful way to get better sleep [26, 27]. Exercises increase the initial temperature of the body and gradually decrease it. As a result, it can affect the reduction of resistance to falling asleep and the reduction of the delay in the onset of sleep. One of the primary reasons for improving sleep habits after physical activity is the physical fatigue caused by exercising. Also, the increase in sleep waves is directly related to the brain’s energy metabolism, which decreases during sleep. An increase in physical activity leads to biological and biochemical changes, such as a shift in the body’s central temperature and an impact on the quality of sleep by stimulating the anterior hypothalamus. In addition, changes in hormonal levels caused by physical activity, including melatonin, cytokines, prolactin, and D2 prostaglandin, have a positive effect on sleep quality [28, 29]. Exercises play an important role in regulating circadian rhythms and improving sleep quality by releasing serotonin. The effect of exercise on increasing brain serotonin concentration has been reported in some studies [30, 31]. Also, serotonin released in the diencephalon and cerebrum can have a positive effect on improving sleep quality and sleeping habits in people [32, 33].

The results related to the subscale of delay in starting to fall asleep showed that the score of this subscale decreased after the intervention. That is, from the point of view of mothers, the time for children to fall asleep has improved after performing sports exercises. The results of Liu et al.‘s study also showed that physical activity improves sleep efficiency, delay in sleep onset, and wake-up time after sleep onset. According to the results of another study, there is a significant relationship between sports training and a shorter sleep delay [34]. Regular participation in sports activities has a positive effect on sleep time. People who exercise regularly experience shorter sleep latency compared to people who do not exercise regularly [35].

According to another finding of the present study, the average score of sleep duration in intervention group children improved significantly. In this regard, the results of studies indicate that regular exercise has a significant impact on increasing the duration of sleep [36, 37]. Inadequate and short sleep has become a common aspect of modern lifestyles across all age groups [38, 39]. Furthermore, the results of a systematic review indicate that individuals with type 1 diabetes may experience poor sleep quality and irregular sleep duration [40]. Physical fatigue and mental happiness caused by exercise [41] can physically and mentally put a person in a condition to have enough sleep during the night.

Based on the results of our study, the average score of sleep anxiety in the children of the experimental group after the intervention was significantly lower than that of the children in the control group. According to the results of studies, physical activity reduces anxiety, reduces symptoms of depression, and improves sleep quality [42, 43]. Insufficient sleep duration increases oxidative stress in the brain and anxiety-like behaviors [44]. Regular exercise plays an effective role in increasing brain-derived neurotrophic factor (BDNF). Increasing this factor can increase neural flexibility, growth, and differentiation of neurons. Therefore, participating in sports exercises can be an effective solution in relieving sleep anxiety [45].

According to mothers’ reports, the average score of frequent night awakenings in the experimental group after the intervention was lower than the control group. Williams et al. also showed in their study that physical activity may lead to better sleep rather than more sleep [46]. The results of a study conducted on children aged 8 to 13 showed that 12.2% of children woke up more than once at night. Therefore, physical activity may be one of the most effective solutions to improve sleep and reduce frequent awakenings at night [47].

The average score of parasomnia in the children of the experimental group after the intervention was significantly lower than that of the children in the control group. Parasomnia, insomnia, and narcolepsy are sleep disorders in children and the understanding of their neurophysiology is evolving [48]. In the literature review, no study was found on the effect of exercise on parasomnia. Considering non-pharmacological techniques, such as behavioral approaches, have been proposed for treating parasomnia, using exercise as a complementary and non-pharmacological treatment may be effective.

The average score of respiratory disorder in the children of the experimental group was lower than that of the children in the control group, but this finding was not statistically significant. Sleep-disordered breathing is associated with an increased risk of cardiovascular disease and mortality. Some studies indicate that the effect of exercise on sleep-disordered breathing is unknown. However, the effect of diet and weight loss on reducing this disorder has been shown in studies [49]. Awad et al. showed that exercise is associated with a reduction in the incidence of sleep-disordered breathing and that reducing physical activity can lead to worsening of sleep-disordered breathing [49]. The results of a meta-analysis also showed that exercise reduces the severity of sleep apnea [50]. In general, it can be said that sports and physical activity are related to sleep-breathing disorders.

The average score of daily sleepiness in children of the experimental group improved significantly after the intervention. The findings of a study showed that exercise is one of the factors that can prevent daytime sleepiness in children. This study showed that children who have daytime sleepiness do less physical activity compared to other children. Therefore, there may be a bidirectional relationship between exercise and daily sleepiness. Severe daytime sleepiness can lead to reduced physical activity levels. Also, people who are physically active during the day show less sleepiness. Designing an exercise program and presenting it to the parents of children referring to medical centers can be effective in managing diabetes and improving the quality of children’s sleep. The program designed in this study can be used as a guideline. According to the findings of this study, it is suggested that doctors, nurses, and treatment staff pay serious attention to the importance of exercise as a non-pharmacological treatment in improving the sleeping habits of diabetic children.

The present study faced limitations in some areas. One of the limitations in the implementation process was related to the insufficient cooperation of children and their parents with the researcher. For this purpose, an effort was made to clearly explain the purpose of the study to them. Also, mothers were assured that their child’s information would be used only for research purposes. The data collection tool was a self-report questionnaire. Therefore, mothers may not have answered the items carefully enough. We suggest that researchers use objective methods such as polysomnography in future studies. The study sample included only boys and girls with type 1 diabetes as participants; therefore, the findings cannot be generalized to other populations.

## Conclusion

The results of this study showed that exercise has a positive and significant effect on improving sleep habits in children with T1D. Experimental evidence suggests that sleep disorders can be one of the problems of diabetic children. Sports exercises as a non-drug treatment can be taken seriously as an effective and joyful solution for children. This effective solution can have a reassuring function both in managing diabetes and improving sleeping habits. Exercises should be designed and implemented based on the recommendations of sports experts and detailed sports protocols. In this regard, it is suggested to engage in aerobic sports with moderate intensity for 2 to 4 times a week (for 40 to 60 min each time), brisk walking, and participation in recreational sports. Paying attention to this important issue improves the effectiveness of sports training.

## Data Availability

The data are available from the corresponding author upon request.

## Abbreviations

CSHQ: Children’s Sleep Habits Questionnaire
T1D: Type 1 diabetes
BDNF: Brain-derived neurotrophic factor
